# Chemical Constituents from *Andrographis echioides* and Their Anti-Inflammatory Activity

**DOI:** 10.3390/ijms14010496

**Published:** 2012-12-27

**Authors:** De-Yang Shen, Shin-Hun Juang, Ping-Chung Kuo, Guan-Jhong Huang, Yu-Yi Chan, Amooru G. Damu, Tian-Shung Wu

**Affiliations:** 1 Department of Chemistry, National Cheng Kung University, Tainan 70101, Taiwan; E-Mail: l3895113@mail.ncku.edu.tw (D.-Y.S.); agdamu@yogivemanauniversity.ac.in (A.G.D.); 2 Graduate Institute of Pharmaceutical Chemistry, China Medical University, No. 91, Hsueh-Shih Road, Taichung 40402, Taiwan; E-Mail: paul@mail.cmu.edu.tw; 3 Department of Biotechnology, National Formosa University, Yunlin 632, Taiwan; E-Mail: pcckuoo@sunws.nfu.edu.tw; 4 Department of Pharmacy, China Medical University, Taichung 40402, Taiwan; E-Mail: gjhuang@mail.cmu.edu.tw; 5 Department of Biotechnology, Southern Taiwan University of Science and Technology, Tainan 71005, Taiwan; E-Mail: yuyichan@mail.stust.edu.tw; 6 Chinese Medicine Research and Development Center, China Medical University Hospital, Taichung 40402, Taiwan

**Keywords:** *Andrographis echioides*, flavonoid, anti-inflammatory, iNOS

## Abstract

Phytochemical investigation of the whole plants of *Andrographis echioides* afforded two new 2′-oxygenated flavonoids (**1)** and (**2**), two new phenyl glycosides (**3**) and (**4**), along with 37 known structures. The structures of new compounds were elucidated by spectral analysis and chemical transformation studies. Among the isolated compounds, (**1**–**2**) and (**6**–**19**) were subjected into the examination for their iNOS inhibitory bioactivity. The structure-activity relationships of the flavonoids for their inhibition of NO production were also discussed.

## 1. Introduction

*Andrographis* (Acanthaceae) is a genus of about 40 species, various members of which have a reputation in indigenous medicine. In traditional Indian medicine, several *Andrographis* species have been used in the treatment of dyspepsia, influenza, malaria and respiratory infections, and as astringent and antidote for poisonous stings of some insects [1,2]. More than 20 species of *Andrographis* have been reported to occur in India. The phytochemistry of this genus has been investigated quite well in view of its importance in Indian traditional medicine and reported to contain several flavonoids [3,4] and labdane diterpenoids [5–10]. *A. echioides*, an annual herb occurring in South India, is listed in the Indian Materia Medica used as a remedy for fevers. However, information on the chemical composition and bioactivity of this species is very rare. There is only report of flavonoids as major components from the extracts of *A. echioides* in the previous literature [11–14]. As part of our program to study the bioactive constituents from *Andrographis* species [15,16], we have investigated the whole plant of *A. echioides* and four new compounds (**1**–**4**) were characterized. Herein, we wish to report on the structure elucidations of compounds **1**–**5** and the effects of flavonoids on NO inhibition in LPS-activated mouse peritoneal macrophages.

## 2. Results and Discussion

### 2.1. Purification and Characterization

The 85% aqueous MeOH extract of the whole plant of *A. echioides* was suspended in H_2_O and partitioned with CHCl_3_ to afford CHCl_3_ and H_2_O soluble layers, respectively. Each layer was subjected into purification by a combination of conventional chromatographic techniques to result in four new compounds (**1**–**4**). In addition, 37 known compounds were identified to be 2′,6′-dihydroxyacetophenone 2′-*O*-β-d-glucopyranoside (**5**) [17], echioidinin 5-*O*-β-d-glucopyranoside (**6**) [15], echioidinin (**7**) [13], pinostrobin (**8**) [18], andrographidine C (**9**) [19], dihydroechioidinin (**10**) [13], tectochrysin 5-glucoside (**11**) [20], methyl salicylate glucoside (**12**) [21], 7,8-dimethoxy-5-hydroxyflavone (**13**) [22], 5,7,8-trimethoxyflavone (**14**) [23], skullcapflavone I 2′-methyl ether (**15**) [13], acetophenone-2-*O*-β-d-glucopyranoside (**16**) [24], androechin (**17**) [14], skullcapflavone I 2′-*O*-β-d-glucopyranoside (**18**) [13], tectochrysin (**19**) [25], 5,7,2′-trimethoxyflavone [26], echioidin [13], skullcapflavone I [27], 5,7-dimethoxyflavone [28], negletein 6-*O*-β-d-glucopyranoside [29], andrographidine E [19], 4-hydroxy-3-methoxy-*trans*-cinnamic acid methyl ester [30], 4-hydroxybenzaldehyde [31], 4-hydroxy-*trans*-cinnamic acid methyl ester [32], *O*-coumaric acid [33], 2,6-dihydroxybenzoic acid [34], 13^2^-hydroxy-(13^2^-*R*)-phaeophytin [35], (*E*)-phytyl-epoxide [36], phytol [37], phytene 1,2-diol [38], (+)-dehydrovomifoliol [39], 3β-hydroxy-5α,6α,-epoxy-7-megastigmen-9-one [40], β-sitosterol [41], β-sitosteryl-3-*O*-β-glucopyranoside [42], squalene [43], 1*H*-indole-3-carbaldehyde [44], and loliolide [45] by comparison of their physical and spectral data with those reported in the literature.

### 2.2. Structural Elucidation of Compounds **1**–**5**

**Figure 1:**
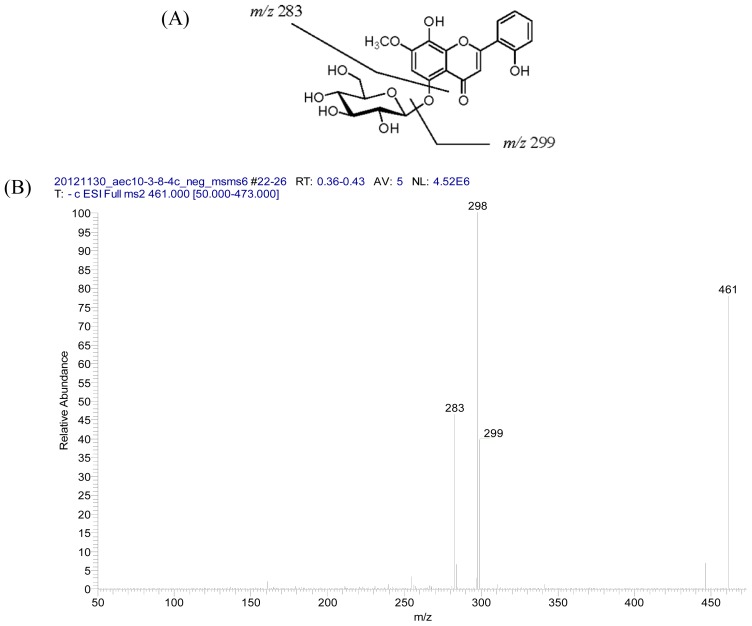
(**A**) Structure of **1** and main fragments under LC/MS/MS (negative ESI); (**B**) LC/MS/MS spectrum.

**Figure 2:**
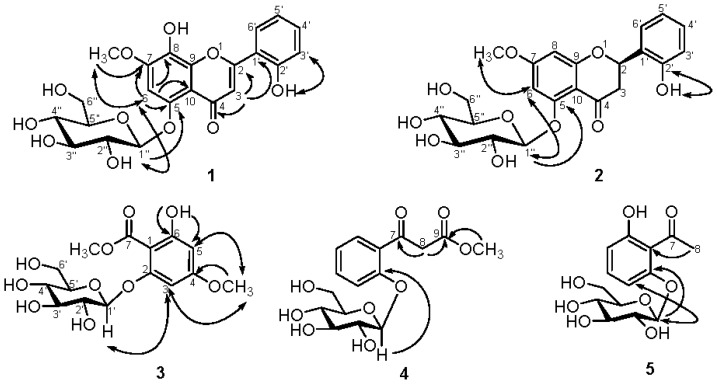
Selected HMBC (→) and NOESY (→) spectrum for compounds **1**–**5**.

Compound **1** was obtained as optically active white amorphous powder. The HRFABMS of **1** showed a molecular ion peak at *m*/*z* 462.1159 corresponding to the molecular formula C_22_H_22_O_11_ and was also corroborated by ^13^C NMR spectrum (Table 1) which displayed 22 carbon signals. A fragment ion at *m*/*z* 301 [(M−162)+H]^+^ observed in the FAB-MS spectrum was the indication of the presence for an *O*-glycosidic hexose moiety. It was also confirmed by *m*/*z* 299 [(M−162)−H]^−^ observed in the negative ESI LC/MS/MS (Figure 1). The UV spectrum exhibited absorption maxima at 271 and 328 nm was typical of the occurrence for the basic skeleton of flavone with 5,7,8-trioxygenation [46]. Addition of sodium acetate did not cause any change in the absorption maximum band II and it suggested the absence of free hydroxyl at C-7. The IR spectrum of **1** showed a hydroxyl absorption band at 3368 cm^−1^ and a carbonyl absorption band at 1628 cm^−1^, respectively. In the ^1^H NMR spectrum (Table 1) of **1**, there were two broad singlets at δ 10.75 and 9.30 exchangeable with D_2_O to be assigned as non-chelated hydroxyl groups at C-2′ and C-8. It also displayed the characteristic aromatic proton signals at δ 8.00, 7.38, 7.04 and 6.99 corresponding for a 2′-oxygenated ring B pattern [19] which was assigned to the H-6′, H-4′, H-3′, and H-5′, respectively. Two sharp singlets at δ 7.17 and 7.05 were attributed to H-6 and H-3 [47], since the ^2^*J*, ^3^*J*-correlations from H-6 to C-5, C-7, C-8 and C-10 and from H-3 to C-2, C-4, and C-1′, respectively, were exhibited in the HMBC spectrum (Figure 2). In addition, a methoxy signal at δ 3.90 (s) which displayed HMBC correlation with C-7 was located at C-7. An anomeric proton signal at δ 4.60 (d, 1H, *J* = 7.6 Hz) suggested the presence of a sugar residue with β-configuration. With the aid of ^13^C NMR spectral analysis, six carbon signals at δ 105.5, 77.8, 76.1, 73.8, 70.4, and 61.3 were identified as d-glucose. The glucose residue in **1** was found to be linked to C-5 since a NOE cross-peak was observed between H-1″ and H-6 in its ROESY spectrum and a ^3^*J*-correlation between H-1″ and C-5 was also displayed in its HMBC spectrum (Figure 2). Acid hydrolysis of **1** with 2N HCl afforded glucose and an aglycone identified as 2′,5,8-trihydroxy-7-methoxyflavone [48]. On the basis of the above spectral evidences, the structure of **1** was established as 5,8,2′-trihydroxy-7-methoxyflavone-5-*O*-β-d-glucopyranoside, and given the trivial name as androgechoside A.

**Figure 3:**
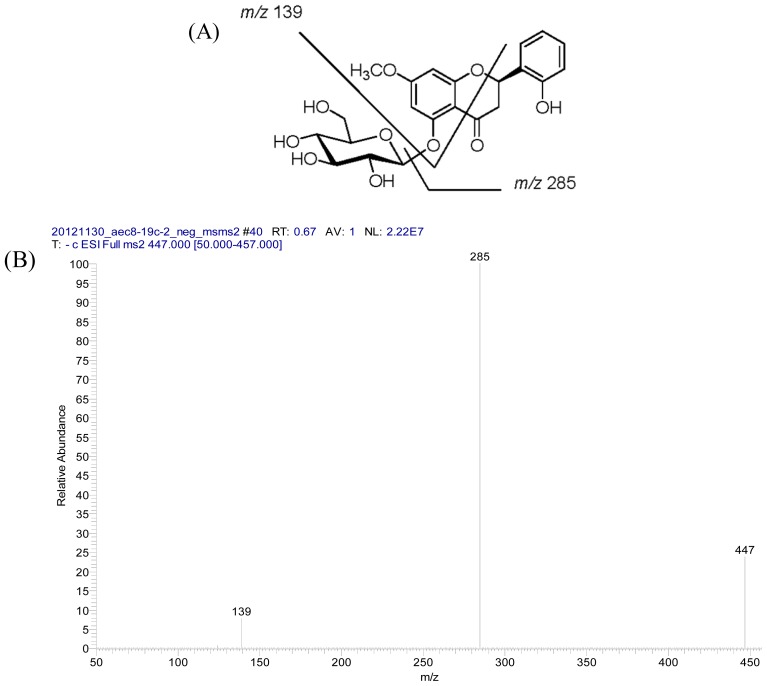
(**A**) Structure of **2** and main fragments under LC/MS/MS (negative ESI); (**B**) LC/MS/MS spectrum.

Compound **2** was purified as optically active white amorphous powder with elemental composition C_22_H_24_O_10_ from its HRFABMS data (*m*/*z* 449.1449 [M+H]^+^). A fragment ion at *m*/*z* 285 [(M−162)−H]^−^ observed in the negative ESI MS/MS spectrum (Figure 3) was the indication of the presence for an *O*-glycosidic hexose moiety. The IR spectrum exhibited absorption bands at 3373 and 1609 cm^−1^ characteristic for the hydroxyl and conjugated carbonyl groups, respectively, together with the UV absorption maximum at 280 nm, suggested the presence of a flavanone skeleton [49]. The UV absorption maximum unaffected by the addition of NaOAc indicated the absence of free hydroxyls at C-7 and C-5 positions. The ^1^H NMR spectrum (Table 1) of **2** exhibited a broad singlet at δ 9.80 exchangeable with D_2_O attributed to a non-chelated hydroxyl group at C-2′ as it showed long range HMBC correlations with these carbons at C-1′ (δ 125.0) and C-2′ (δ 154.3) (Figure 2). A typical ABCD coupled system at δ 6.85, 7.17, 6.84 and 7.41 established the presence of four adjacent aromatic protons (H-3′, H-4′, H-5′ and H-6′) in ring B and also supported the presence of OH-2′. In addition, two *meta* coupled aromatic doublets at δ 6.32 (*J* = 2.5 Hz) and 6.46 (*J* = 2.5 Hz) attributed for H-8 and H-6, respectively, suggested that compound **2** possessed flavanone basic skeleton. In the upfield region, three sets of doublets of doublets at δ 5.70 (1H, dd, *J* = 12.5, 3.0 Hz), 3.04 (1H, dd, *J* = 16.0, 12.5 Hz) and 2.64 (1H, dd, *J* = 16.0, 3.0 Hz) which were characteristic signals of H-2, H-3_eq_ and H-3_ax_ of flavanone also supported this suggestion. Moreover, a methoxy signal at δ 3.79 (s) which displayed NOESY correlations with H-6 and H-8 was deduced to be located at C-7. The appearance of one glucose moiety in **2** was revealed by the proton signals at δ 4.83 (1H, d, *J* = 7.5 Hz) and 3.26–3.43 (5H, m), and the carbon resonances at δ 101.9, 77.6, 76.4, 73.5, 70.0, and 61.0 [50]. The glucose should attach at C-5 as β-configuration, which were identified by the coupling constant of the anomeric proton and the ^3^*J*-HMBC correlation between H-1″ (δ 4.83) and C-5 (δ 159.7) (Figure 2). The location of β-glucose was further confirmed through the NOE crosspeak between H-1″ and H-6. The circular dichroism (CD) spectrum of **2** showed a negative Cotton effect at 337 nm and a positive Cotton effect at 275 nm, suggesting the absolute configuration at C-2 to be *R* [51]. On the basis of the foregoing studies, the structure of **2** was determined as (2*R*)-5,2′-dihydroxy-7- methoxyflavanone-5-*O*-β-d-glucopyranoside and trivially named as androgechoside B.

**Figure 4:**
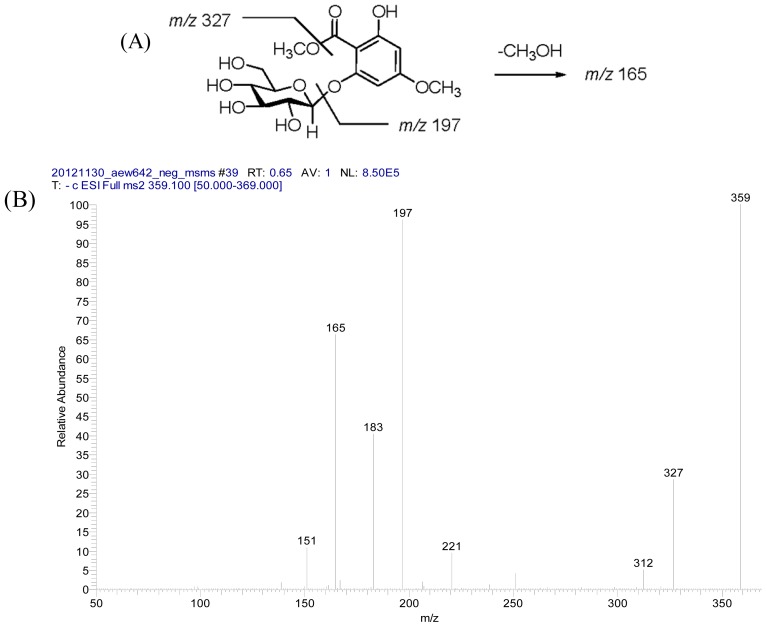
(**A**) Structure of **3** and main fragments under LC/MS/MS (negative ESI); (**B**) LC/MS/MS spectrum.

Compound **3** was obtained as an optically active white amorphous powder. Its molecular formula, C_15_H_20_O_10_, was established on the basis of HRESIMS (*m*/*z* 383.0952 [M+Na]^+^, calcd 383.0954). A fragment ion at *m*/*z* 197 [(M−162)−H]^−^ observed in the negative ESI MS/MS spectrum (Figure 4). The IR spectrum of **3** sh Darmstadt owed absorption bands for hydroxyl and carbonyl groups at 3449 and 1652 cm^−1^. The ^1^H NMR spectrum (Table 2) of **3** displayed an intramolecular hydrogen bonding proton signal at δ 11.4 (s), two *meta* coupled aromatic protons at δ 6.14 (d, *J* = 2.4 Hz) and 6.36 (d, *J* = 2.4 Hz), and two methoxy groups at δ 3.81 (s) and 3.88 (s), respectively. In addition, the proton signals for an anomeric proton at δ 4.96 (d, *J* = 7.6 Hz), oxygenated methylene at δ 3.91 (1H, m) and 3.69 (1H, m), and oxygenated methines at δ 3.60–3.40 (4H, m) suggested the presence of one sugar moiety. In the ^13^C NMR spectrum (Table 2) of **3**, the carbon resonances at δ 102.5, 74.7, 77.7, 71.2, 78.0, 62.6 [50] further confirmed that **3** was substituted with one glucose. Moreover, in the ^13^C NMR spectrum six characteristic aromatic carbons at δ 166.1, 165.2, 161.0, 98.5, 96.1 and 95.4 indicated the occurrence of an unsymmetrically substituted phloroglucinol unit [52]. Acid hydrolysis of **3** produced d-glucose and its absolute configuration was determined by HPLC method. The relative locations of the methyl ester, hydroxyl, methoxy group and sugar moieties were established from HMBC spectrum (Figure 2), in which correlations of the methoxy group (δ 3.81) with C-4 (δ 166.1), the hydroxyl group (δ 11.4) with C-6 (δ 165.2) and C-5 (δ 96.1) were observed. The location of sugar unit was assigned by an NOESY experiment, in which NOESY correlation was found between δ 4.96 (1H, d, *J* = 7.6 Hz) and δ 6.36 (1H, d, *J* = 2.4 Hz), indicating that the glucose was attached at C-2 through oxygen atom. Thus, the structure of compound **3** was determined as 2-*O*-β-d-glucopyranosyl-4-methoxy-2,4, 6-trihydroxybenzoate and named trivially as androechioside A.

**Figure 5:**
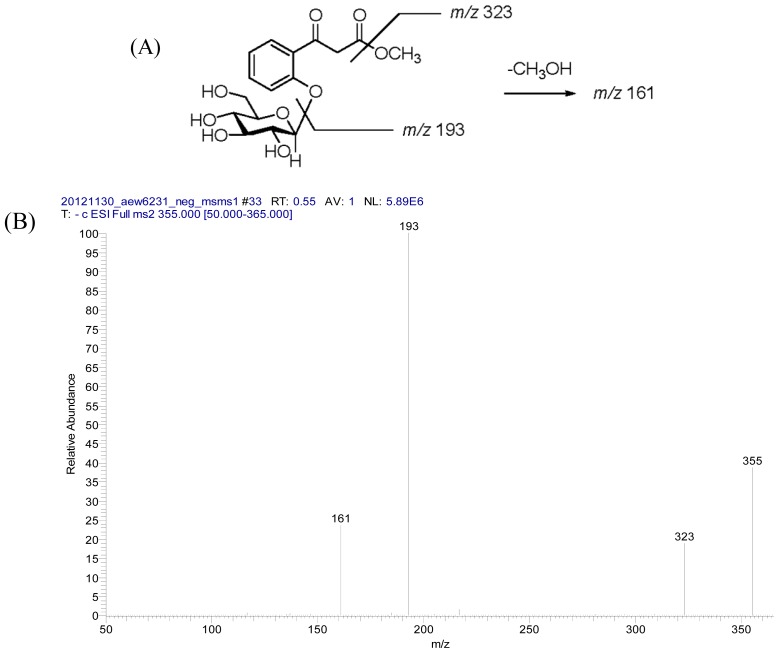
(**A**) Structure of **4** and main fragments under LC/MS/MS (negative ESI); (**B**) LC/MS/MS spectrum.

Compound **4** was purified as optically active white amorphous powder and the molecular formula was determined as C_16_H_20_O_9_ by HR-ESI mass spectrometric analysis. A fragment ion at *m/z* 193 [(M−162)−H]^−^ observed in the negative ESI MS/MS spectrum (Figure 5). The IR spectrum showed hydroxyl, ester, and carbonyl groups at 3381, 1731, and 1672 cm^−1^, respectively. The ^1^H NMR spectroscopic data (Table 2) showed the characteristic of 1,2-disubstituted aromatic ring system from the chemical shifts at δ 7.12 (1H, dd, *J* = 7.8, 7.8 Hz), 7.33 (1H, d, *J* = 7.8 Hz), 7.54 (1H, dd, *J* = 7.8, 7.8 Hz), and 7.75 (1H, d, *J* = 7.8 Hz). It also showed that the compound had a methyl ester at δ 3.66 (3H, s), and suggested a β-keto ester structure for it with an unsubstituted α-methylene group [δ 4.23 (1H, d, *J* = 16.4 Hz) and 4.07 (1H, d, *J* = 16.4 Hz)]. Furthermore, the ^1^H and ^13^C NMR spectroscopic data (Table 2) showed the presence of a β-glucopyranosyl unit including the anomeric proton signal at δ 5.15 (1H, d, *J* = 7.2 Hz) and the carbon signals at δ 101.1, 77.0, 77.0, 73.4, 70.1 and 61.4, in addition to the signals of the aglycone moiety. The location of sugar unit was assigned by an NOESY experiment, in which NOESY correlation was found between H-1′ (δ 5.15) and H-3 (δ 7.33), indicating that the glucose was attached at C-2. Moreover, HMBC spectrum (Figure 2) further confirmed the linkage of sugar unit from the ^3^*J*-correlation between H-1′ (δ 5.15) and C-2 (δ 156.9). Therefore, the chemical structure of **4** was elucidated as methyl 3-(2-hydroxyphenyl)-3-oxopropanoate 2-*O*-β-d-glucopyranoside and named trivially as androechioside B following the convention.

Compound **5** was afforded as optically active white amorphous powder with the assistance of conventional chromatographic methods. It possessed a molecular formula C_14_H_18_O_8_ deduced from the HR-ESI-MS (*m*/*z* 337.0897 [M+H]^+^, calcd 337.0899). The IR spectrum showed the absorption bands at 3409 and 1628 cm^−1^ attributed to the presence of hydroxyl and conjugated carbonyl groups. The ^1^H NMR spectrum (Table 2) of **5** displayed a broad hydroxyl group at δ 13.0 (1H, br s), three mutually coupled aromatic protons signals at δ 6.55 (1H, dd, *J* = 8.4, 1.2 Hz), 6.75 (1H, dd, *J* = 8.4, 1.2 Hz) and 7.38 (1H, dd, *J* = 8.4, 8.4 Hz), and one methyl singlet at δ 2.77 (3H). In addition, the HMQC correlation between the methyl singlet (δ 2.77) and a deshielded carbon at δ 33.8 suggested it to be an acetyl group. Moreover, an anomeric proton at δ 5.12 (1H, d, *J* = 8.8 Hz), together with a set of signals arising from a sugar moiety at δ 101.2, 77.3, 77.1, 73.6, 70.2 and 61.6 in its ^13^C NMR spectrum, revealed the presence of one glucose fragment. Acidic hydrolysis of compound **5** liberated d-glucose which was determined by comparison with the authentic sample with HPLC method. The locations of the glucosyl, acetyl and hydroxyl groups were determined by 2D spectral experiments. In the HMBC spectrum (Figure 2) of **5**, the methyl protons (δ 2.77) and the anomeric proton (δ 5.12) were correlated with C-1 (δ 111.8) and C-2 (δ 159.5), respectively. Furthermore, the NOESY correlation of anomeric proton (δ 5.12) with H-3 (δ 6.75) confirmed the structure of **5**. Compound **5** was therefore determined to be 2′,6′-dihydroxyacetophenone 2′-*O*-β-d-glucopyranoside. It has previously only been prepared by synthesis [17] and is reported herein from nature for the first time.

### 2.3. Anti-Inflammatory Activity

Inflammation is related to morbidity and mortality of many diseases and is recognized as part of the complex biological response of vascular tissues to harmful stimuli. It is the host response to infection or injury, which involves the recruitment of leukocytes and the release of inflammatory mediators, including nitric oxide (NO). NO is the metabolic by-product of the conversion of l-arginine to l-citrulline by a class of enzymes termed NO synthases (NOS). Numerous cytokines can induce the transcription of inducible NO synthase (iNOS) in leukocytes, fibroblasts, and other cell types, accounting for enhanced levels of NO. In the experimental model of acute inflammation, inhibition of iNOS can have a dose-dependent protective effect, suggesting that NO promotes edema and vascular permeability. NO also has a detrimental effect in chronic models of arthritis, whereas protection is seen with iNOS inhibitors. The iNOS inhibiting potentials of **1**–**2** and **6**–**19** were evaluated by examining their effects on LPS-induced iNOS-dependent NO production in RAW 264.7 cells determined by MTT assays. Cells cultured with **1**–**2** and **6**–**19** at different concentrations except **18** (at 42 μM) used in the presence of 100 ng/mL LPS for 24 h did not change cell viability thus the NO inhibiting effects may not due to the cytotoxicity (Table 3). In the examined concentration ranges (5.25–74 μM), NO production decreased in the presence of **1**–**2** and **6**–**19** in a dose-dependent manner (Table 3). Flavonoids are widely distributed in the higher plants capable of modulating the activity of enzymes and affect the behavior of many cell systems, including NO inhibitory activity. The structure-activity relationships of 3′,4′-oxygenated flavones were discussed by Matsuda [53] and Kim *et al.* [54]. In 1999, Kim *et al.* [54] examined the naturally occurred flavonoids for NO production inhibitory activity in LPS-activated RAW 264.7 cells and the following structural requirements were afforded: (a) the strongly active flavonoids possessed the C2–C3 double bond and 5,7-dihydroxyl groups; (b) the 8-methoxyl group and 4′- or 3′,4′-vicinal substitutions favorably affected inhibitory activity; (c) the 2′,4′-(*meta*)-hydroxyl substitutions abolished the inhibitory activity; (d) the 3-hydroxyl moiety reduced the activity; (e) flavonoid glycosides were not active regardless of the types of aglycones. *Andrographis* species are noted for profuse production of 2′-oxygenated flavones and in the present study, the bioactive data of the examined flavonoids using RAW 264.7 cells were in agreement with the previous report by Kim *et al.*, and the additional structural requirements of flavonoids for NO production inhibitory activity were suggested as follows: (1) the glycosidic moiety reduced the activity, like **9** and **14**; (2) the 2′-hydroxyl group did not cause significant effects on NO inhibitory activity; (3) methylation of 5-hydroxyl group enhanced the activity, like **13** and **14** (Table 4). The structure-activity relationships of flavonoids for NO production inhibitory activity resulted from our study clarified the insufficiency in the previous report.

## 3. Experimental Section

### 3.1. General

The UV spectra were obtained with Hitachi UV-3210 spectrophotometer. The IR spectra were measured with a Shimadzu FTIR Prestige-21 spectrometer. Optical rotations were recorded with a Jasco DIP-370 digital polarimeter in a 0.5 dm cell. The ESIMS and HRESIMS were taken on a Bruker Daltonics APEX II 30e spectrometer. The FABMS and HRFABMS were taken on a Jeol JMS-700 spectrometer. The ESIMS (negative ESI) data were measured using a Thermo TSQ Quantum Ultra LC/MS/MS spectrometer. The ^1^H and ^13^C NMR spectrums were measured by Bruker Avance 300, 400 and AV-500 NMR spectrometers with TMS as the internal reference, and chemical shifts are expressed in δ (ppm). The CD spectrum was recorded in a Jasco J-720 spectrometer. Sephadex LH-20, silica gel (70–230 and 230–400 mesh; Merck, Darmstadt, Germany) and reversed-phase silica gel (RP-18; particle size 20–40 μm; Silicycle) were used for column chromatography, and silica gel 60 F_254_ (Merck, Darmstadt, Germany) and RP-18 F_254S_ (Merck, Darmstadt, Germany) were used for TLC. HPLC was performed on a Shimadzu LC-10AT_VP_ (Tokyo, Japan) system equipped with a Shimadzu SPD-M20A diode array detector at 250 nm, a Purospher STAR RP-8e column (5 μm, 250 × 4.6 mm) and Cosmosil 5C_18_ ARII (250 × 4.6 mm i.d. Nacalai Tesque Inc.) (Tokyo, Japan). LPS (endotoxin from *Escherichia coli*, serotype 0127:B8), MTT (3-[4,5-dimethylthiazol-2-yl]-2,5-diphenyltetrazolium bromide) and other chemicals were purchased from Sigma Chemical Co. (St. Louis, MO, USA).

### 3.2. Plant Materials

The whole plant of *A. echioides* Nees was collected from Tirupati, Andhra Pradesh, India in May 1998. The plant was authenticated by Professor C. S. Kuoh, Department of Life Science, National Cheng Kung University, Taiwan. The voucher specimens (DG-199) have been deposited in the herbarium of the Department of Botany, Sri Venkateswara University, Tirupati, India; and Department of Chemistry, National Cheng Kung University, Tainan, Taiwan, respectively.

### 3.3. Extraction and Isolation

The plant materials (10 kg) were cut into small pieces and heated at refluxed with 85% aqueous MeOH (5 × 80 L). The resulting MeOH extract (704 g) was partitioned between CHCl_3_ and H_2_O (each 3 L) for five times to yield the CHCl_3_ layer (208 g) and H_2_O layer (446 g). The CHCl_3_ layer was subjected to silica gel column chromatography (CC) using a gradient mixture of CHCl_3_-MeOH (19:1, 9:1, 7:1, 5:1, 3:1, 1:1) as eluent to give 11 fractions (Fr. 1–11). Fr. 2 was purified by CC over silica gel (*n*-hexane–diisopropyl, ether 40:1) to yield squalene (74.6 mg). Fr. 4 was separated by CC over silica gel (*n*-hexane–acetone, 49:1) to yield pinostrobin (67.2 mg). Fr. 5 was subjected to chromatography on silica gel (*n*-hexane–EtOAc, 9:1) to yield tectochrysin (20.3 mg). Fr. 6 was chromatographed over silica gel (*n*-hexane–acetone, 3:1) to yield dihydroechioidinin (11.2 mg), tectochrysin (111.9 mg) and skullcapflavone I 2′-methyl ether (76.7 mg). Fr. 7 was purified by CC over silica gel (*n*-hexane–acetone, 3:1) to yield eight fractions: 7.1, 7.2, 7.3, 7.4, 7.5, 7.6, 7.7 and 7.8. Fr. 7.3 was chromatographed over silica gel (*n*-hexane–acetone, 3:1) to yield 7,8-dimethoxy-5-hydroxyflavone (35.8 mg), 5,7,2′-trimethoxyflavone (6.5 mg) and echioidinin (6.3 mg). Fr. 7.4 was chromatographed over silica gel (*n*-hexane–diisopropyl ether, 3:1) to yield skullcapflavone I (6.2 mg) and dihydroechioidinin (1.2 mg). Fr. 7.5 was chromatographed over silica gel (*n*-hexane–EtOAc, 3:1) to yield β-sitosterol (33.2 mg), androgechoside B (1.3 mg), tectochrysin (12.3 mg), 5,7,8-trimethoxyflavone (30.7 mg) and 5,7-dimethoxyflavone (7.6 mg). Fr. 7.6 was chromatographed over silica gel (*n*-hexane–EtOAc, 2:1) to yield 4-hydroxy-3-methoxy-*trans*-cinnamic acid methyl ester (5.6 mg), 4-hydroxybenzaldehyde (7.2 mg) and 13^2^-hydroxy-(13^2^-*R*)-phaeophytin (2.4 mg). Fr. 7.7 was chromatographed over silica gel (CHCl_3_–acetone, 29:1) to yield (*E*)-phytyl epoxide (11.5 mg), phytol (27.9 mg), dehydrovomifoliol (5.3 mg) and 3β-hydroxy-5α,6α-epoxy-7-megastigmen-9-one (5.1 mg). Fr. 7.8 was chromatographed over silica gel (CHCl_3_–MeOH, 39:1) to yield 1*H*-indole-3-carbaldehyde (2.3 mg), loliolide (10.1 mg) and phytene 1,2-diol (11.5 mg). Fr. 8 was chromatographed over silica gel (CHCl_3_–MeOH, 7:1) to yield 7,8-dimethoxy-5-hydorxyflavone (3.3 mg), skullcapflavone I 2′-*O*-β-d-glucopyranoside (4.5 mg), echioidinin 5-*O*-β-d-glucopyranoside (1.2 mg), dihydroechioidinin (677.6 mg), androechin (7.3 mg), androgechoside B (7.9 mg) and tectochrysin 5-glucoside (5.0 mg). Fr. 9 was chromatographed over silica gel (EtOAc–MeOH, 9:1) to yield andrographidine E (10.1 mg), androechin (1.2 mg) and β-sitosteryl-3-*O*-β-glucopyranoside (25.3 mg). Fr. 10 was separated by CC over silica gel (CHCl_3_–MeOH, 5:1) to yield echioidin (5.1 mg), androgechoside B (0.5 mg), negletein 6-*O*-β-d-glucopyranoside (3.8 mg), androgechoside A (34.7 mg) and 4-hydorxy-*trans*-cinnamic acid methyl ester (4.0 mg).

The H_2_O layer (446 g) was separated on Diaion HP-20 (0%–100% MeOH) to yield seven fractions. Fr. 3 was purified by CC over Sephadex LH-20 (0%–100% MeOH) to yield 2,6-dihydroxybenzoic acid (8.2 mg). Fr. 4 was subjected to CC over Sephadex LH-20 (0%–100% MeOH) to yield *O*-coumaric acid (3.4 mg). Fr. 5 was separated on Sephadex LH-20 (0%–100% MeOH) to yield androechioside B (18.9 mg) and androechioside D (12.0 mg). Fr. 6 was separated on Sephadex LH-20 (0%–100% MeOH) to yield methyl salicylate glucoside (2.1 mg), androechioside A (3.4 mg) and acetophenone-2-*O*-β-d-glucopyranoside (17.0 mg). Fr. 7 was subjected to CC over Sephadex LH-20 (0%–100% MeOH) to yield andrographidine C (26.9 mg), echioidinin 5-*O*-β-d-glucopyranoside (3.2 mg) and androechin (0.9 mg).

#### 3.3.1. Androgechoside A (**1**)

White amorphous powder; [α]_D_^25^ − 166.7 (*c* 0.04, MeOH); UV (MeOH), λ_max_ (log ɛ) 328 (3.61), 271, (3.92), 224 (3.75), 207 (4.05) nm; IR (KBr) *v*_max_ 3368, 1628, 1573, 1076, 1049 cm^−1; 1^H and ^13^C NMR see Table 1; FABMS (positive mode) *m*/*z* (rel. int.): 463 [M+H]^+^; HRFABMS *m*/*z*: 462.1159 [M]^+^.

#### 3.3.2. Androgechoside B (**2**)

White amorphous powder; [α]_D_^25^ − 149.2 (*c* 0.02, MeOH); UV (MeOH), λ_max_ (log ɛ) 383 (3.83), 280 (4.38), 211 (4.53), 204 (4.57) nm; CD (MeOH): nm λ_max_ (Δɛ) 337 (−4.92), 275 (+15.5); IR (KBr) *v*_max_ 3373, 1609, 1272, 1070, 1034 cm^−1; 1^H and ^13^C NMR see Table 1; FABMS (positive mode) *m*/*z* (rel. int.): 449 [M+H]^+^; HRFABMS *m/z*: 449.1449 [M+H]^+^ (calcd 449.1448).

#### 3.3.3. Androechioside A (**3**)

White amorphous powder; [α]_D_^25^ + 4.5 (*c* 0.68, MeOH); UV (MeOH), λ_max_ (logɛ) 263 (3.26), 218 (3.45), 203 (3.84); IR (KBr) *v*_max_ 3449, 1652, 1615, 1514, 1327, 1160, 1072 cm^−1; 1^H and ^13^C NMR see Table 2; ESIMS *m/z* (rel. int.): 383 [M+Na]^+^; HRESIMS *m/z*: 383.0952 [M+Na]^+^ (calcd for C_15_H_20_O_10_Na, 383.0954).

#### 3.3.4. Androechioside B (**4**)

White amorphous powder; [α]_D_^25^ − 27.8 (*c* 0.34, MeOH); UV (MeOH), λ_max_ (log ɛ) 301 (3.83), 246 (4.10), 210 (4.23) nm; IR (KBr) *v*_max_ 3381, 1731, 1672, 1598, 1231, 1072 cm^−1; 1^H and ^13^C NMR see Table 2; ESIMS *m/z* (rel. int.): 379 [M+Na]^+^; HRESIMS *m/z*: 379.1007 [M+Na]^+^ (calcd for C_16_H_20_O_9_Na, 379.1005).

#### 3.3.5. 2′,6′-Dihydroxyacetophenone 2′-*O*-β-d-glucopyranoside (**5**)

White amorphous powder; [α]_D_^25^ − 8.8 (*c* 0.12, MeOH); UV (MeOH), λ_max_ (log ɛ) 331 (3.23), 265 (3.48), 214 (3.61), 209 (4.12) nm; IR (KBr) *v*_max_ 3409, 1628, 1600, 1456, 1075 cm^−1; 1^H and ^13^C NMR see Table 2; ESIMS *m/z* (rel. int.): 337 [M+Na]^+^; HRESIMS *m/z*: 337.0897 [M+Na]^+^ (calcd for C_14_H_18_O_8_Na, 337.0899).

### 3.4. Determination of Aldose Configuration

Compounds **1**–**5** (each 0.5 mg) were hydrolyzed with 0.5M HCl (0.4 mL) in a screw-capped vial at 60 °C for 1 h. The reaction mixture was neutralized with Amberlite IRA400 and filtered. The filtrates were dried in vacuo, then dissolved in 0.1 mL of pyridine containing l-cysteine methyl ester (0.5 mg), and reacted at 60 °C for 1 h. To those mixtures were added a solution of *O*-tolylisothiocyanate in pyridine (5 mg/1 mL) at room temperature for 1 h. Those reaction mixtures were directly analyzed by HPLC (Cosmosil 5C_18_ ARII (250 × 4.6 mm i.d. Nacalai Tesque Inc., Tokyo, Japan); 20% CH_3_CN in 50 mM acetate; flow rate 0.8 mL/min; detection, 250 nm). d-glucose (t*_R_* 40.5 min) was identified as the sugar moieties of **1**–**5** based on comparisons with authentic samples of d-glucose (t*_R_* 40.5 min).

### 3.5. Determination of iNOS Inhibitory Effects

#### 3.5.1. Cell Culture

A murine macrophage cell line RAW264.7 (BCRC No. 60001) was purchased from the Bioresources Collection and Research Center (BCRC) of the Food Industry Research and Development Institute (Hsinchu, Taiwan). Cells were cultured in plastic dishes containing Dulbecco’s Modified Eagle Medium (DMEM, Sigma, St. Louis, MO, USA) supplemented with 10% fetal bovine serum (FBS, Sigma, St. Louis, MO, USA) in a CO_2_ incubator (5% CO_2_ in air) at 37 °C and subcultured every 3 days at a dilution of 1:5 using 0.05% trypsin-0.02% EDTA in Ca^2+^-, Mg^2+^-free phosphate-buffered saline (DPBS).

#### 3.5.2. Cell Viability

Cells (2 × 10^5^) were cultured in 96-well plate containing DMEM supplemented with 10% FBS for 1 day to become nearly confluent. Then cells were cultured with samples in the presence of 100 ng/mL LPS for 24 h. After that, the cells were washed twice with DPBS and incubated with 100 μL of 0.5 mg/mL MTT for 2 h at 37 °C testing for cell viability. The medium was then discarded and 100 μL dimethyl sulfoxide (DMSO) was added. After 30-min incubation, absorbance at 570 nm was read using a microplate reader (Molecular Devices, Orleans Drive, Sunnyvale, CA, USA).

#### 3.5.3. Measurement of Nitric Oxide/Nitrite

NO production was indirectly assessed by measuring the nitrite levels in the cultured media and serum determined by a colorimetric method based on the Griess reaction [55]. The cells were incubated with a test sample in the presence of LPS (100 ng/mL) at 37 °C for 24 h. Then, cells were dispensed into 96-well plates, and 100 μL of each supernatant was mixed with the same volume of Griess reagent (1% sulfanilamide, 0.1% naphthyl ethylenediamine dihydrochloride, and 5% phosphoric acid) and incubated at room temperature for 10 min, the absorbance was measured at 540 nm with a Micro-Reader (Molecular Devices, Orleans Drive, Sunnyvale, CA, USA). By using sodium nitrite to generate a standard curve, the concentration of nitrite was measured form absorbance at 540 nm.

#### 3.5.4. Statistical Analysis

Experimental results were presented as the mean ± standard deviation (SD) of three parallel measurements. IC_50_ values were estimated using a non-linear regression algorithm (SigmaPlot 8.0; SPSS Inc. Chicago, IL, USA). Statistical significance is expressed as * *p* < 0.05, ** *p* < 0.01, and *** *p* < 0.001.

## 4. Conclusions

In the previous literature, there are four *Andrographis* species containing diterpenoids such as andrographolide, including *A. paniculata*, *A. affinis*, *A. lineata*, and *A. wightiana*. In our investigation, the major constituents of the titled plant were flavonoids rather than the crystalline bitter principle analogous to diterpenoids. In the evaluation of NO inhibition activity, compounds **10** and **14** were the most effective and the IC_50_ values were 37.6 ± 1.2 μM and 39.1 ± 1.3 μM, respectively. These results suggested that the *Andrographis* species are valuable sources for the discovery of natural anti-inflammatory lead drugs.

## Figures and Tables

**Table 1:** ^1^H and ^13^C NMR data of compounds **1** and **2**^a^ in DMSO-*d*_6_.

	1		2	

Position	δ_H_ (*J*, Hz)	δ_C_	δ_H_ (*J*, Hz)	δ_C_
2		159.1	5.70, dd (12.5, 3.0)	74.2
3	7.05, s	111.5	2.64, dd (16.0, 3.0)	43.9
			3.04, dd (16.0, 12.5)	
4		178.3		189.6
5		149.5		159.7
6	7.17, s	102.1	6.46, d (2.5)	97.7
7		151.6		165.4
8		130.9	6.32, d (2.5)	95.9
9		146.1		164.3
10		109.5		106.3
1′		117.1		125.0
2′		156.8		154.3
3′	7.04, d (8.0)	117.1	6.85, d (6.5)	115.6
4′	7.38, dd (8.0, 8.0)	132.7	7.17, dd (6.5, 6.5)	129.4
5′	6.99, ddd (8.0, 8.0, 1.6)	119.6	6.84, dd (6.5, 6.5)	119.2
6′	8.00, dd (8.0, 1.6)	128.7	7.41, d (6.5)	126.9
1″	4.60, d (7.6)	105.5	4.83, d (7.5)	101.9
2″	3.27–3.37, m	73.8	3.26–3.43, m	73.5
3″	3.27–3.37, m	77.8	3.15, m	76.4
4″	3.13, m	70.4	3.26–3.43, m	70.0
5″	3.27–3.37, m	76.1	3.26–3.43, m	77.6
6″	3.46, m	61.3	3.26–3.43, m	61.0
	3.73, m		3.64, m	
OH-2′^b^	10.75, br s		9.80, br s	
OH-8^b^	9.30, br s			
OCH_3_-7	3.90, s	56.3	3.79, s	55.9

a 1 H NMR data were measured at 400 MHz for **1**, and 500MHz for **2**; ^13^C NMR data were measured at 100 MHz for **1**, and 125 MHz for **2**. The assignments are based on DEPT, ^1^H–^1^H COSY, HMQC and HMBC spectra.

b D_2_O exchangeable.

**Table 2:** ^1^H and ^13^C NMR Data for **3**–**5** in acetone-*d*_6_.

Position	3^a^		4^b^		5^a^	

	δ^C^	δ^H^ mult (*J*, Hz)	δ^C^	δ^H^ mult (*J*, Hz)	δ^C^	δ^H^ mult (*J*, Hz)
1	98.5		127.3		111.8	
2	161.0		156.9		159.5	
3	95.4	6.36, d (2.4)	116.0	7.33, d (7.8)	105.3	6.75, dd (8.4, 1.2)
4	166.1		134.3	7.54, dd (7.8, 7.8)	136.1	7.38, dd (8.4, 8.4)
5	96.1	6.14, d (2.4)	122.0	7.12, dd (7.8, 7.8)	111.2	6.55, dd (8.4, 1.2)
6	165.2		129.9	7.75, d (7.8)	163.8	
7	171.7		193.7		205.0	
8			49.8	4.23, d (16.4)	33.8	2.77, s
				4.07, d (16.4)		
9			168.5			
1′	102.5	4.96, d (7.6)	101.1	5.15, d ( 7.2)	101.2	5.12, d (8.8)
2′	74.7	3.60–3.40, m	73.4	3.60–3.40, m	73.6	3.59, dd ( 8.8, 8.8)
3′	77.7	3.60–3.40, m	77.0	3.60–3.40, m	77.3	3.46, dd ( 8.8, 8.8)
4′	71.2	3.60–3.40, m	70.1	3.60–3.40, m	70.2	3.54, dd (8.8, 8.8)
5′	78.0	3.60–3.40, m	77.0	3.60–3.40, m	77.1	3.55, m
6′a	62.6	3.91, m	61.4	3.87, m	61.6	3.88, dd (12.0, 2.0)
6′b		3.69, m		3.72, m		3.70, dd (12.0, 5.2)
OCH_3_-4	55.9	3.81, s				
OCH_3_-7	52.5	3.88, s				
OCH_3_-9			51.1	3.66, s		
OH-6		11.4, s				13.0, br s

a Data were measured at 400MHz (^1^H) and 125 (^13^C);

b Data were and in 300MHz (^1^H) and 75MHz (^13^C).

**Table 3:** Effects of **1**–**2** and **6**–**19** on lipopolysaccharide (LPS)-induced cell viability and NO production of RAW 264.7 macrophages ║.

	Dose (μM)	Cell viability (% of control)	NO level	NO inhibition (% of control)	IC_50_ (μM)
control	(−)	100.0 ± 3.3	0.2 ± 0.9	(−)	
LPS	(+)	96.9 ± 6.1	59.4 ± 0.8 ###	(−)	
**1**	5.25	92.1 ± 4.2	48.1 ± 1.5	19.0 ± 2.5	>42
	10.5	86.6 ± 1.5	48.1 ± 1.7	19.1 ± 2.9	
	21	83.9 ± 3.5	45.3 ± 1.4	23.7 ± 2.3	
	42	80.1 ± 3.7	42.7 ± 1.1 *	28.1 ± 1.8	

**2**	5.63	103.6 ± 2.3	49.8 ± 4.6	16.1 ± 7.7	>45
	11.25	99.2 ± 6.8	46.2 ± 2.5	22.2 ± 4.2	
	22.5	99.2 ± 4.2	41.8 ± 1.6 *	29.6 ± 2.7	
	45	99.8 ± 6.0	39.9 ± 0.7 **	32.8 ± 1.1	

**6**	5.63	92.6 ± 3.0	45.0 ± 2.7	24.2 ± 4.6	>45
	11.25	85.7 ± 4.6	46.2 ± 1.8	22.3 ± 3.0	
	22.5	84.6 ± 3.5	44.4 ± 2.1 *	25.2 ± 3.5	
	45	81.4 ± 2.1	43.2 ± 1.8 *	27.2 ± 3.0	

**7**	8.75	90.5 ± 4.0	45.2 ± 1.2	23.9 ± 2.0	>70
	17.5	88.2 ± 1.7	44.6 ± 2.6	25.0 ± 4.3	
	35	84.9 ± 2.2	43.8 ± 2.1 *	26.2 ± 3.5	
	70	83.2 ± 5.6	43.5 ± 2.7 *	26.8 ± 4.6	

**8**	9.25	90.1 ± 7.0	51.7 ± 1.9	12.9 ± 3.2	>74
	18.5	86.4 ± 6.5	47.2 ± 2.1	20.5 ± 3.6	
	37	82.0 ± 4.4	47.1 ± 1.8	20.7 ± 3.1	
	74	79.3 ± 1.7	42.2 ± 2.5 *	28.9 ± 4.1	

**9**	5.5	89.7 ± 2.7	42.7 ± 4.0 *	28.1 ± 6.8	>44
	11	84.8 ± 1.8	41.1 ± 1.6 *	30.8 ± 2.7	
	22	83.4 ± 1.9	38.7 ± 3.0 **	34.8 ± 5.0	
	44	80.2 ± 1.7	38.3 ± 2.0 **	35.6 ± 3.3	

**10**	8.75	89.4 ± 8.3	41.3 ± 1.3	30.4 ± 2.3	37.6 ± 1.2
	17.5	84.9 ± 2.0	39.6 ± 1.5 **	33.3 ± 2.5	
	35	82.1 ± 3.7	30.7 ± 1.5 **	48.3 ± 2.6	
	70	80.1 ± 2.9	21.2 ± 2.7 ***	64.2 ± 4.5	

**11**	5.75	98.9 ± 4.4	50.6 ± 0.6	14.9 ± 1.0	>46
	11.5	96.2 ± 4.2	47.5 ± 4.0	20.0 ± 6.7	
	23	96.5 ± 4.5	46.7 ± 0.9	21.4 ± 1.4	
	46	93.0 ± 3.4	46.5 ± 1.9	21.7 ± 3.2	

**12**	8	100 ± 5.4	50.0 ± 2.3	15.8 ± 3.9	>64
	16	96.5 ± 9.2	49.5 ± 1.0	16.7 ± 1.7	
	32	96.3 ± 4.2	48.0 ± 1.0	19.2 ± 1.6	
	64	94.3 ± 2.1	45.5 ± 2.0	23.4 ± 3.4	

**13**	8.5	98.1 ± 6.6	52.2 ± 1.3	12.1 ± 2.2	>68
	17	97.7 ± 1.7	48.8 ± 0.7	17.8 ± 1.3	
	34	90.8 ± 2.9	47.9 ± 3.9	19.4 ± 6.6	
	68	90.8 ± 8.5	47.5 ± 1.0	20.1 ± 1.7	

**14**	8	98.9 ± 3.5	40.8 ± 2.7 *	31.4 ± 4.5	39.1 ± 1.3
	16	97.9 ± 3.8	34.3 ± 2.0 **	42.3 ± 3.4	
	32	94.3 ± 4.6	32.2 ± 1.5 **	45.8 ± 7.5	
	64	92.2 ± 3.2	23.1 ± 2.3 ***	61.1 ± 3.8	

**15**	7.5	99.4 ± 4.3	45.6 ± 1.1	23.3 ± 1.8	>60
	15	99.2 ± 2.9	43.0 ± 3.5 *	27.7 ± 5.9	
	30	98.9 ± 1.8	42.0 ± 4.0 *	29.3 ± 6.7	
	60	98.8 ± 6.9	41.2 ± 1.9 *	30.6 ± 3.1	

**16**	8.5	101.5 ± 4.3	56.5 ± 2.0	4.9 ± 3.4	>68
	17	100.3 ± 1.0	51.7 ± 2.6	13.0 ± 4.4	
	34	99.6 ± 1.0	50.4 ± 4.6	15.1 ± 7.8	
	68	93.7 ± 0.9	44.6 ± 0.5 *	24.9 ± 0.9	

**17**	5.63	100.8 ± 0.8	52.4 ± 2.9	11.8 ± 4.9	>45
	11.25	98.8 ± 3.5	51.7 ± 4.6	12.9 ± 7.7	
	22.5	97.8 ± 3.6	50.8 ± 3.8	14.5 ± 6.4	
	45	98.3 ± 1.9	46.4 ± 4.0	21.8 ± 6.7	

**18**	5.25	101.4 ± 1.2	53.5 ± 4.4	9.9 ± 7.4	>42
	10.5	100.5 ± 1.8	52.1 ± 3.7	12.3 ± 6.2	
	21	95.6 ± 0.8	50.7 ± 4.5	14.6 ± 7.6	
	42	74.1 ± 6.6	(−)	(−)	

**19**	9.25	103.5 ± 4.8	52.7 ± 4.7	11.2 ± 8.0	>74
	18.5	98.2 ± 11.3	47.5 ± 4.8	20.1 ± 8.0	
	37	92.9 ± 7.6	46.7 ± 4.0	21.4 ± 6.8	
	74	81.6 ± 6.5	44.4 ± 2.8 *	25.2 ± 4.7	

║ The data were presented as mean ± S.D. for three different experiments performed in triplicate.

### compared with sample of control group.

* *p* < 0.05,

** *p* < 0.01, and

*** *p* < 0.001 were compared with LPS-alone group.

**Table 4:** Effects of flavones and flavanones on NO production inhibitory activity in LPS-activated mouse peritoneal macrophages.

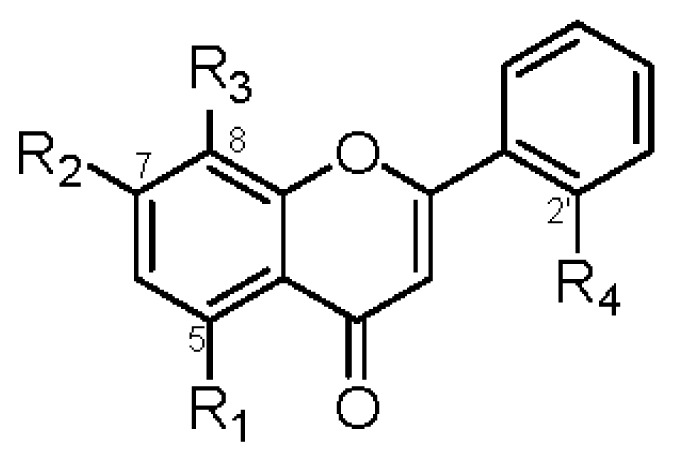						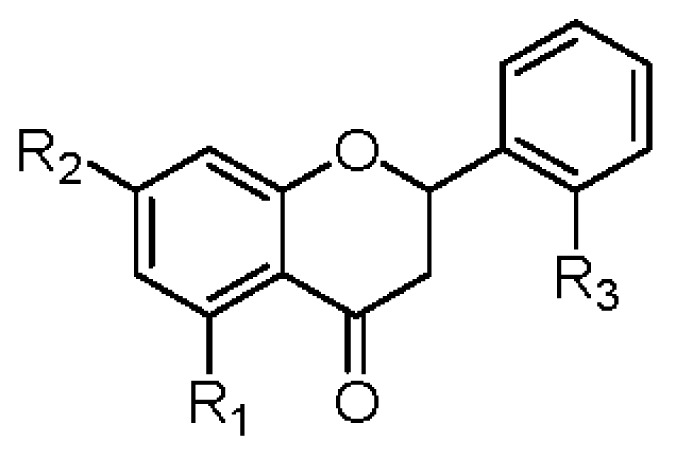				
	R_1_	R_2_	R_3_	R_4_	IC_50_ (μM)		R_1_	R_2_	R_3_	IC_50_ (μM)
**1**	OGlc	OCH_3_	OH	OH	>42	**2**	OGlc	OCH_3_	OH	>45
**6**	OGlc	OCH_3_	H	OH	>45	**8**	OH	OCH_3_	H	>74
**7**	OH	OCH_3_	H	OH	>70	**10**	OH	OCH_3_	OH	37.6 ± 1.2 _b_
**9**	OGlc	OCH_3_	OCH_3_	H	>44					
**11**	OGlc	OCH_3_	H	H	>46					
**13**	OH	OCH_3_	OCH_3_	H	>64					
**14**	OCH_3_	OCH_3_	OCH_3_	H	39.1 ± 1.3 a					
**15**	OH	OCH_3_	OCH_3_	OCH_3_	>60					
**18**	OH	OCH_3_	OCH_3_	OGlc	>42					
**19**	OH	OCH_3_	H	H	>74					

Glc, β-d-glucopyranosyl;

a Values in parentheses represent the inhibition (%) at 8 μM;

b Values in parentheses represent the inhibition (%) at 8.75 μM.
